# EBV-Driven HLH and T Cell Lymphoma in a Child with X-Linked Agammaglobulinemia: A Genetically Confirmed Case Report and Literature Review

**DOI:** 10.3390/jpm15080365

**Published:** 2025-08-09

**Authors:** Jose Humberto Perez-Olais, Elizabeth Mendoza-Coronel, Jose Javier Moreno-Ortega, Jesús Aguirre-Hernández, Gabriela López-Herrera, Marco Antonio Yamazaki-Nakashimada, Patricia Baeza-Capetillo, Guadalupe Fernanda Godínez-Zamora, Omar Josue Saucedo-Ramírez, Laura C. Bonifaz, Ezequiel M. Fuentes-Pananá

**Affiliations:** 1 Unidad de Investigación en Virología y Cáncer, Hospital Infantil de México “Federico Gómez”, México City 06720, Mexico; jholais@gmail.com (J.H.P.-O.); elizabeth.mendozacoronel@gmail.com (E.M.-C.); jose.moreno68@anahuac.mx (J.J.M.-O.); 2 Laboratorio de Genómica, Genética y Bioinformática, Hospital Infantil de México “Federico Gómez”, México City 06720, Mexicopatricia.baezac@cetis57.edu.mx (P.B.-C.); lggb@himfg.edu.mx (G.F.G.-Z.); 3 Laboratorio de Inmunodeficiencias, Instituto Nacional de Pediatría, México City 04530, Mexico; gablopezh@pediatria.gob.mx; 4 Departamento de Inmunología Clínica, Instituto Nacional de Pediatría, México City 04530, Mexico; myamazakin@pediatria.gob.mx; 5 Departamento de Alergia e Inmunología Pediátrica, Hospital Infantil de México “Federico Gómez”, México City 06720, Mexico; dr.omar.saucedo@gmail.com; 6 Coordinación de Investigación en Salud, Centro Médico Nacional Siglo XXI, Instituto Mexicano del Seguro Social, México City 06720, Mexico

**Keywords:** X-linked agammaglobulinemia, Epstein–Barr virus, *BTK* mutation, hemophagocytic lymphohistiocytosis, T cell lymphoma, inborn errors of immunity

## Abstract

**Introduction**: X-linked agammaglobulinemia (XLA) is a prototypical inborn error of immunity (IEI) caused by mutations in the *BTK* gene, leading to a profound deficiency of mature B cells and severe pan-hypogammaglobulinemia. The Epstein-Barr virus (EBV), which primarily infects B lymphocytes, is believed to be unable to establish persistence in these patients due to the lack of its natural reservoir. Indeed, current evidence supports that EBV infection is typically refractory in individuals with XLA. **Methods**: We describe the clinical and molecular characterization of a 10-year-old male patient with genetically confirmed XLA who developed EBV viremia, hemophagocytic lymphohistiocytosis (HLH), and EBV-positive cutaneous T cell lymphoma. Diagnosis was supported by flow cytometry, serology, quantitative PCR, EBER *in situ* hybridization, histopathology, and whole-exome sequencing. **Results**: Despite the complete absence of peripheral B cells, EBV was detected in leukocytes and multiple tissues, indicating active infection. The patient developed HLH and a T cell lymphoma with EBER-positive infiltrates. Genetic analysis revealed a nonsense mutation in *BTK* (1558C>T, R520*), confirming XLA. The clinical course included multiple episodes of neutropenia, viral and bacterial infections, and severe systemic inflammation. **Conclusions**: This is the first documented case of an XLA patient with confirmed *BTK* mutation presenting with clinical features more consistent with chronic active EBV infection. These findings challenge the prevailing paradigm that XLA confers protection against EBV-related diseases and further support the possibility of EBV noncanonical reservoirs leading to immune dysregulation. EBV should also be considered in the differential diagnosis of XLA patients presenting with systemic inflammation or lymphoproliferative disease.

## 1. Introduction

Inborn errors of immunity (IEI) are a group of genetic disorders that impair the development, function, and/or number of immune cells. With 500+ defective genes identified to date, these disorders display considerable clinical and immunological heterogeneity, ranging from severe combined immunodeficiency, where patients lack a functional immune system, to milder forms that may go unnoticed until later in life. Depending on the affected gene, IEIs can compromise nearly all immune cell lineages or selectively impair lymphoid, myeloid, or even a single immune cell type, such as B cells [1].

XLA is caused by mutations in the *BTK* gene, located on the X chromosome. BTK contains multiple conserved domains that mediate its function in B cell signaling. The pleckstrin homology (PH) domain allows membrane localization through binding to PIP3. The TEC homology (TH) domain contributes to protein stability via a zinc finger motif. The SH3 and SH2 domains mediate protein–protein interactions, recognizing proline-rich sequences and phosphotyrosine motifs, respectively. Together, these domains regulate BTK activation and its downstream signaling essential for the development of B lymphocytes. BTK deficiency arrests their maturation at the pre-B cell stage in the bone marrow, preventing the formation of functional peripheral mature B cells [2,3,4,5,6]. Consequently, XLA patients exhibit profoundly reduced peripheral blood B cell counts (CD19+ cells < 2%) and severely reduced or absent immunoglobulin levels due to the lack of antibody-producing plasma cells [7,8]. XLA is one of the most common IEIs, with affected individuals primarily suffering from recurrent pyogenic infections such as pneumonia, conjunctivitis, otitis media, and bacteremia [9].

**Epstein-Barr virus (EBV)**, discovered in 1964 by Anthony Epstein, Bert Achong, and Yvonne Barr, represents the first human oncogenic virus to be identified. EBV is a ubiquitous human herpesvirus, infecting approximately 95% of the world’s population [10,11,12]. During its infection cycle, EBV enters the host through the lymphoepithelial tissue of the oropharynx, primarily targeting B cells, where it establishes latent, lifelong infection within the memory B cell compartment. Reactivation from latency leads to viral replication and shedding, with newly formed virions infecting additional B lymphocytes or being transmitted to new hosts via the oral cavity. EBV is associated with a wide spectrum of malignancies, including B cell, T cell, and NK cell lymphomas, as well as gastric and nasopharyngeal carcinomas. EBV is also implicated in severe inflammatory and autoimmune diseases [13]. Despite its pathogenic potential, most individuals maintain EBV in control, exhibiting no obvious clinical symptoms.

There is a subset of IEIs characterized by a loss of immune control over EBV infection, leading to high circulating viral loads and severe, often life-threatening complications, in which malignancy, inflammation, and autoimmunity often can coexist. These IEIs generally affect the function of cytotoxic T cells and/or NK cells, the primary immune effectors responsible for EBV control [14]. Given that EBV resides in B cells, conditions that impair B cell development, such as XLA, are generally thought to protect against EBV-driven diseases by eliminating its natural reservoir. Nevertheless, EBV-related disease has been previously reported in patients with hypogammaglobulinemia. For instance, Kleinman et al. described an EBV-positive mucocutaneous ulcer in a patient with low levels of all immunoglobulins, yet with normal B cell numbers [15]. Interestingly, a previous study conducted on XLA patients concluded that they are refractory to EBV infection, proposing that their markedly low B cell numbers prevent EBV infection [16,17,18]. However, such conclusions are not absolute and may be challenged by exceptional cases that help to reveal the full complexity of EBV infection in immunodeficient hosts.

Here, we describe a pediatric patient with XLA caused by a *BTK* mutation who, despite lacking circulating mature B cells, developed active EBV viremia, hemophagocytic lymphohistiocytosis (HLH), and an EBV-positive cutaneous T cell lymphoma. Despite the complete absence of peripheral B cells, EBV was detected in leukocytes and multiple tissues, with EBER-ISH confirming its presence in neoplastic T cells, indicating active infection in noncanonical cellular reservoirs. The clinical and virological features of this case overlap with chronic active EBV (CAEBV) disease, a rare T/NK cell lymphoproliferative syndrome usually seen in individuals with functional immune cells. This report highlights a rare and clinically significant presentation and is, to our knowledge, the first to describe EBV-associated lymphoma and HLH in a patient with genetically confirmed XLA. We further provide a focused review of the literature on EBV-related disease in patients with agammaglobulinemia and IEIs. This manuscript adheres to CARE guidelines (http://www.care-statement.org/ accessed on 17 February 2025) for reporting case reports.

## 2. Materials and Methods

### 2.1. Clinical Evaluation and Record Review

The patient underwent a comprehensive clinical evaluation on admission, including a history of recurrent infections, physical examination, and family history of immunodeficiency. Initial laboratory tests included a complete blood count, leukocyte differential, and serum chemistry. Immunologic testing included quantification of serum immunoglobulin levels (IgG, IgA, IgM, and IgE) by nephelometry and flow cytometry to analyze lymphocyte subsets (CD3+, CD4+, CD8+, CD16+/CD56+, and CD19+ cells).

Additionally, histopathological evaluation of skin biopsies was performed. Hematoxylin and eosin staining was used for general histology. Immunohistochemistry with chromogenic detection was performed using antibodies against CD3, CD4, CD8, CD20, CD30, CD79, and Ki67 to characterize lymphoid phenotype and assess proliferation. Serological testing for EBV antigens (VCA, EA, and EBNA), CMV, HHV-6, HHV-7, and HHV-8 were conducted using standard commercial kits.

Diagnostic criteria for HLH were evaluated according to the HLH-2004 protocol. All patient clinical information, except for EBV viral load detection and EBER-ISH, was obtained from the medical record.

### 2.2. EBV Detection and Viral Load

The viral load of EBV was determined according to the protocol of Morales-Sánchez A et al., 2018 [19]. Briefly, DNA was purified from 1 × 10^6^ PBMCs and 400 µL plasma using the QIAamp DNA Mini Kit (Qiagen, Hilden, Germany) according to the manufacturer’s protocol. The DNA concentration was quantified using a NanoDrop 1000 spectrophotometer (Thermo Fisher Scientific, Waltham, MA, USA), and quality was assessed by optical density (260/280 ratio) and amplification of the endogenous β-actin gene. EBV detection was performed using 100 ng of PBMCs DNA or 5 µL of plasma DNA per qPCR reaction. Results were expressed as viral copies per microgram of DNA or calculated and reported as copies per milliliter of plasma. Quantification was based on standard curves and all samples were analyzed in triplicate.

### 2.3. Genetic Analysis

Genomic DNA was extracted from peripheral blood mononuclear cells as described in the previous section. Whole-exome sequencing was performed to identify pathogenic variants associated with IEI. Bioinformatic analysis included alignment to the GRCh37/hg19 reference genome, variant calling, and annotation using clinically relevant databases. Sanger sequencing and Western blotting of BTK were performed in the index patient and affected relatives.

### 2.4. EBER-Based In Situ Hybridization (EBER-ISH)

Detection of EBV-infected cells in tissue samples was performed by EBER-ISH. Formalin-fixed paraffin-embedded (FFPE) sections from lung, brain, and bone marrow were hybridized with digoxigenin-labeled oligonucleotide probes specific to EBV-encoded small RNAs (EBERs). The hybridization signal was visualized using an anti-digoxigenin antibody and chromogenic detection system. Positive nuclear staining confirmed the presence of EBV-infected cells.

### 2.5. Review of Literature

We searched the PubMed database for the terms (“XLA manifestations”, “CAEBV clinical manifestations” OR “XLA and CAEBV” and we selected articles published in English between 2014 and 2025. We summarized the most relevant clinical features reported for both conditions.

### 2.6. Ethical Compliance

This study was conducted in accordance with the Declaration of Helsinki and approved by the Ethics and Biosafety Committees of the Hospital Infantil de México Federico Gómez (Protocol Registration HIM-2020-017, SSA 1649). Written informed consent was obtained from the patient’s legal guardian for diagnostic and research purposes.

## 3. Results

### 3.1. Case Presentation

A patient was initially admitted at 4 years of age with vesicular dermatosis on the right lower extremity, **progressing** to severe cellulitis accompanied by persistent fever (38.7 °C), limited mobility, and pain. Hematological evaluation revealed pancytopenia, with CD19+ B cell counts at 0.4%, and low levels of neutrophils (neutropenia), eosinophils, and immunoglobulins (Table 1). Initial treatment included Dicloxacillin and monthly subcutaneous immunoglobulin infusions (545 mg/kg). This therapeutic approach resulted in an improvement of the patient’s cutaneous lesion.

A few days later, the patient experienced sudden neurological deterioration and right hemiparesis. Computed tomography (CT) revealed early-stage bilateral cerebritis with left-sided predominance. A fronto-temporo-parietal decompressive craniectomy was performed, resulting in patient improvement. Mydriasis and lesions compatible with varicella (chickenpox) infection were also noted. A subsequent CT scan showed multiple pulmonary lesions and lung collapse (atelectasis) consistent with a fungal infection, which was treated with voriconazole. Bronchoalveolar aspirate cytology revealed epithelial cells, an inflammatory infiltrate with polymorphonuclear cells (PMNs), and bacterial colonies.

Unexplained facial dermal lesions raised suspicion of a lymphoproliferative process, prompting a skin biopsy for histopathologic and immunophenotypic analysis. The biopsy revealed a dense lymphocytic infiltrate and a lymphoproliferative process positive for Ki67, CD3 (100%), CD8 (40%), and CD4 (60.5%), and negative for CD20, CD30, CD19, and CD79 (Table 2). These findings were interpreted as cutaneous T cell lymphoma, and the patient was immediately started on systemic chemotherapy according to the pediatric protocol: intravenous vincristine (VCR; 2 mg/m^2^) and daunorubicin (DNR; 25 mg/m^2^) every seven days for four doses, followed by intravenous etoposide and cytarabine (Ara-C; 300 mg/m^2^) every seven days for two months.

A positron emission tomography (PET) scan showed bilateral cervical lymphadenopathy, mediastinal adenopathy, and infra- and supra-diaphragmatic pulmonary infiltration involving the liver, spleen, and muscle. During chemotherapy, the patient was admitted twice to the emergency department: first for neutropenic colitis and septic shock, and second for papulovesicular lesions on the left forearm and necrotic lesions on the right forearm. A Tzanck test for herpes simplex virus (HSV) was positive, and acyclovir (1500 mg/day) was administered. Subsequent admissions revealed pneumonia caused by bocavirus (HBoV) and respiratory syncytial virus (RSV), along with at least two episodes of febrile neutropenia. The clinical course and therapeutic interventions are summarized in Table 2.

**Figure 1:**
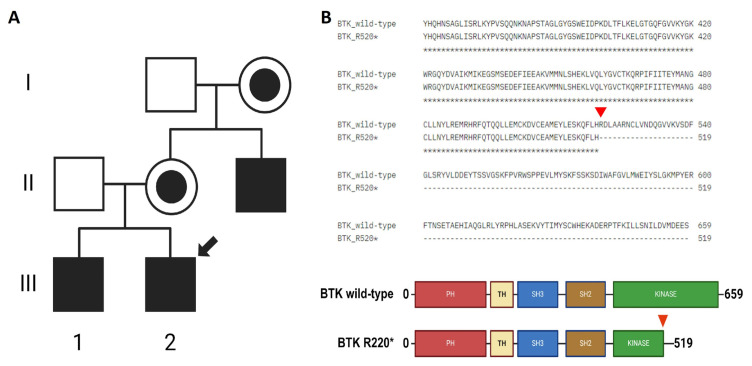
Family background and identification of *BTK* mutation. (**A**) Maternal male relatives have been diagnosed with XLA. Filled squares indicate the affected patients, the one marked with a black arrow indicates the proband, healthy members are indicated by empty squares, and women carriers of the mutation are indicated by black filled circles. (**B**) The 1558C>T changein *BTK* results in a premature stop codon R520* (red arrow).

The patient had a family history of XLA, with an older brother and a maternal uncle also diagnosed with the condition (Figure 1A). A *BTK* mutation was confirmed by whole-exome sequencing (WES) in all three cases and validated by Sanger sequencing and Western blotting for the uncle (Supplementary Figure S1). The sequencing analysis identified a single-nucleotide variant(SNV) (1558C>T) in the three patients, resulting in a premature stop codon (Arg520*) in the kinase domain of the BTK protein (Figure 1B and Supplementary Figure S1). This mutation is expected to impair kinase activity and therefore disrupt BTK function. Importantly, this was the only pathogenic variant of clinical significance detected in our patient, and its classification as pathogenic was confirmed based on available evidence from variant databases such as ClinVar and Franklin, as well as according to the American College of Medical Genetics and Genomics (ACMG) guidelines. This variant was first reported in 1994 during mutational screening of unrelated XLA patients, with two additional unrelated cases described in 1998. Codons 520 and 525 are considered mutational hotspots in the *BTK* gene, with both nonsense and missense variants previously reported [3,4,20]. At the age of 10 years, the patient developed HLH, further complicating the clinical course (Table 2). HLH is a life-threatening hyperinflammatory syndrome characterized by non-remitting fever, hepatosplenomegaly, cytopenias, coagulopathy, lipid abnormalities, and multiple organ failure. It results from immune dysregulation involving cytotoxic T cells, NK cells, and histiocytes. EBV infection has been implicated as a major trigger of HLH [21]. The patient was treated according to the HLH-2004 protocol and included etoposide, dexamethasone and cyclosporine A [7,22,23,24].

**Figure 2:**
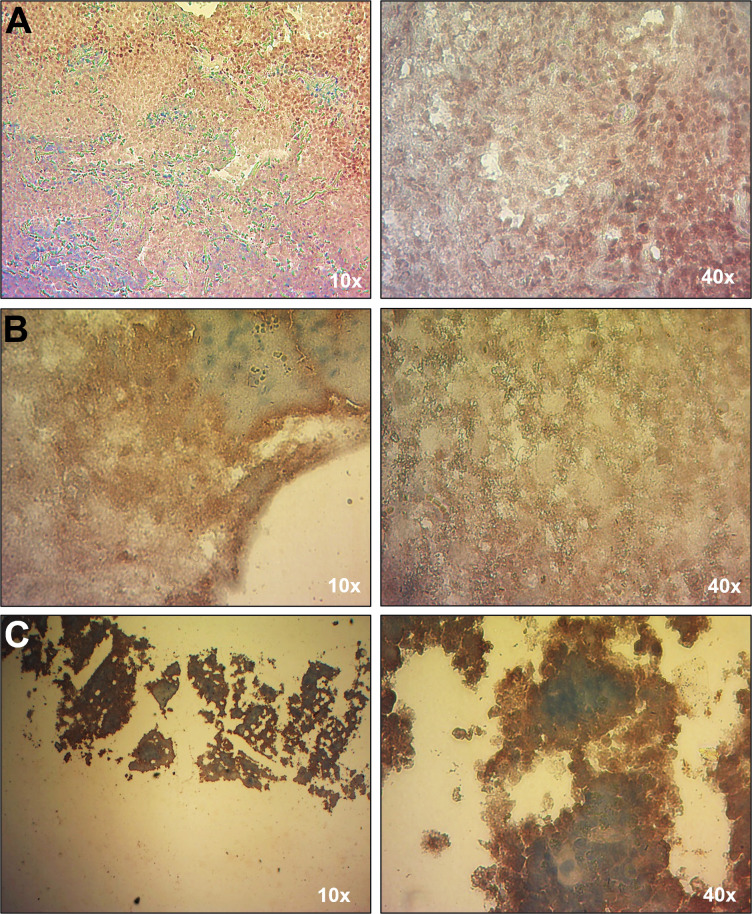
Detection of EBV in tissues with EBER-ISH. An EBER-ISH assay was performed to determine the presence of EBV in the following tissues: (**A**) lung nodule; (**B**) brain parenchyma, and (**C**) bone marrow. All tissues showed a positive signal for EBV (brown signal), indicative of EBV infection.

Due to the patient’s history of immunodeficiency and EBV-associated conditions, including HLH and cutaneous T cell lymphoma, EBV load was analyzed by qPCR. The first test, conducted while the patient was receiving chemotherapy, showed no detectable EBV in whole blood. However, a subsequent EBV load assay performed four years later, coinciding with the onset of HLH, revealed the presence of EBV exclusively in leukocytes (640 copies/µg of DNA), with no detectable virus in plasma. This result was consistent with the observed lymphoproliferative activity. EBV infection was confirmed in multiple tissues with lymphoproliferative infiltrate, including lung nodules, brain parenchyma, and bone marrow, by EBER-based in situ hybridization (EBER-ISH) (Figure 2). Serological tests for EBV lytic (VCA and EAD) and latent (EBNA1) antigens were negative, likely reflecting the absence of plasma cells (Table 1). The patient tested negative for other herpesviruses, including CMV, HHV-6, HHV-7, and HHV-8, by qPCR. The most recent hospital admission of the patient was due to neutropenia and SARS-CoV-2 (COVID-19) infection.

### 3.2. Review of the Literature

XLA and CAEBV are two distinct immunological disorders that differ markedly in genetic origin, immunopathogenesis, and clinical course. XLA is a well-characterized IEI caused by germline mutations in the *BTK* gene, resulting in impaired B cell development, profound hypogammaglobulinemia, and increased susceptibility to bacterial infections, predominantly affecting males and manifesting in early infancy [25]. In contrast, CAEBV is a chronic, EBV-driven lymphoproliferative disorder characterized by persistent infection of T or NK cells [26,27,28]. It is not classified as a primary immunodeficiency per se, but may arise in the context of acquired somatic mutations (e.g., *STAT3*, *DDX3X*, or *BCOR*) or immune dysregulation affecting EBV control. CAEBV affects both sexes and is notably more prevalent in East Asian and Latin American populations, including Mexico, with cases ranging from pediatric to adult at onset.

In XLA, classical manifestations include recurrent sinopulmonary infections such as otitis media, sinusitis, and pneumonia. Gastrointestinal symptoms, including chronic diarrhea and IBD-like enteropathy, are also frequent [29,30,31]. Less common complications include enteroviral meningoencephalitis, pericarditis, or pulmonary alveolar proteinosis [32,33,34]. Additional atypical features reported in case series include allergic rhinitis, Mohr–Tranebjaerg syndrome, and hepatocellular carcinoma [30,31]. In contrast, CAEBV typically manifests with prolonged fever, hepatosplenomegaly, lymphadenopathy, cytopenia, rash, lymphoproliferation, and HLH. NK/T lymphoproliferation and HLH are common and often defining complications. Atypical features include coronary artery aneurysms, generalized myositis, pulmonary hypertension, and CNS involvement [27,35,36,37]. Misdiagnosis is frequently reported due to overlapping symptoms with autoimmune and systemic inflammatory conditions.

XLA tends to manifest in early childhood with infection-driven pathology resulting from severe antibody deficiency, whereas CAEBV is characterized by chronic systemic inflammation and tissue infiltration driven by clonal expansion of EBV-infected T and/or NK cells. HLH is a hallmark of CAEBV, but it is very rare in XLA, where it is typically triggered by bacterial infections [38,39].

#### 3.2.1. Susceptibility to Microbial Infections

XLA patients are highly susceptible to encapsulated bacteria, including *Streptococcus pneumoniae*, *Haemophilus influenzae*, and *Staphylococcus aureus*, due to impaired antibody-mediated opsonization. The broader infectious spectrum includes *Pseudomonas aeruginosa*, *Campylobacter jejuni*, *Salmonella* spp., *Giardia lamblia*, rotavirus, and SARS-CoV-2 [32,38,40,41,42]. In contrast, CAEBV is primarily driven by persistent EBV infection, which sometimes also confers an increased susceptibility to other opportunistic pathogens: viral (CMV, HHV-3, and HHV-6 ), bacterial (e.g., *Pseudomonas* spp.), and fungal (*Candida* spp.). This susceptibility is mainly due to immune dysregulation and cytopenias (Supplementary Tables S1 and S2) [37,38,43,44].

#### 3.2.2. Immunological and Cellular Deficiencies

In XLA, some patients exhibit a “leaky” phenotype, retaining low but detectable B-cell numbers and partial immunoglobulin synthesis. Neutropenia occurs in approximately 10–25% of cases, possibly resulting from defective Fc receptor signaling in BTK-dependent myeloid cells [42,45].

In contrast, CAEBV is characterized by complex and progressive pan-lymphocyte abnormalities, including reduced numbers of CD4+ and CD8+ T cells, B cells, and NK cells. A common immunological feature is the inversion of the CD4/CD8 ratio, observed in 32.1% of adult CAEBV patients in one study [28]. This inversion is often accompanied by concurrent decreases in both CD4+ and CD8+ T cell subsets, reflecting widespread T-cell dysregulation (Supplementary Table S2).

In summary, XLA is a genetically determined, B-cell intrinsic immunodeficiency, whereas CAEBV is driven by chronic immune activation and clonal expansion of EBV-infected T or NK cells.

#### 3.2.3. BTK Mutations and Genotype–Phenotype Correlation

XLA is caused by over 2400 different mutations in the *BTK* gene, including missense, nonsense, and splice-site changes (https://databases.lovd.nl/shared/genes/BTK (accessed on 30 May 2025)). Large deletions involving *BTK* and adjacent genes, such as *TIMM8A*, cause syndromic forms, such as Mohr-Tranebjaerg syndrome [33], which includes neurodegeneration and hearing loss [33].

In contrast, CAEBV involves somatic mutations arising in EBV-infected T or NK cells, often affecting genes such as *DDX3X, TET2,* and *BCOR* [46,47]. Clonal T-cell receptor (TCR) gene rearrangements are also frequently observed, supporting both the diagnosis of CAEBV and the associated risk of lymphoproliferation (Supplementary Table S2) [35,38,46,48,49]. Thus, while XLA is a monogenic, inherited disorder principally affecting bone marrow B cell progenitor cells, CAEBV arises from somatic mutations in mature T and/or NK cells and is more closely related to clonal lymphoproliferative disease.

#### 3.2.4. Atypical Manifestations in CAEBV and XLA Related to EBV

Atypical manifestations in XLA include inflammatory enteropathy, pulmonary conditions such as alveolar proteinosis, Mohr–Tranebjaerg syndrome, and HLH-like episodes mainly triggered by bacterial infection [30,31,32,34]. In CAEBV, atypical presentations include cerebral aneurysm rupture, systemic myositis, and gastrointestinal perforation [35,36,37,50]. Additionally, several reports describe cytokine storm-like syndromes and macrophage hyperactivation as central features of disease severity. Elevated inflammatory cytokines, particularly in the context of activated CD8+ T cells and persistent EBV infection, have been implicated in systemic inflammation and multiorgan damage [26,28,43,45].

Both XLA and CAEBV disorders display an aggressive, potentially fatal course if untreated. However, XLA morbidity is manageable by treating recurrent infections and regular immunoglobulin replacement therapy, in contrast to CAEBV, where hematopoietic stem cell transplantation (HSCT) offers curative potential.

#### 3.2.5. Differential Biomarker Patterns in CAEBV and XLA

Laboratory findings clearly delineate the distinct pathophysiological profiles of CAEBV and XLA. Patients with CAEBV consistently exhibit elevated liver transaminases (ALT and AST), indicative of hepatic inflammation or multi-organ involvement. Ferritin levels, a surrogate marker of inflammation and macrophage activation, are also profoundly elevated in CAEBV. Other laboratory abnormalities include markedly elevated soluble IL-2 receptor alpha (sIL-2Rα), hypertriglyceridemia, increased lactate dehydrogenase (LDH), and inversion of the CD4/CD8 ratio, which together support a hyperinflammatory state and lymphoproliferative activity (Supplementary Table S3) [51].

In contrast, patients with XLA typically display profound B cell lymphopenia and pan-hypogammaglobulinemia. Unlike CAEBV, where vaccine responses can range from partial to intact, XLA patients consistently fail to mount effective humoral responses owing to their intrinsic B cell deficiency. Notably, as CAEBV progresses, gradual B cell depletion and waning antibody titers have also been observed in advanced stages of disease.

## 4. Discussion

The interaction between EBV and IEI has been widely explored in the context of cytotoxic T cell and NK cell deficiencies [13,14,21,24]. However, the relationship between EBV and humoral immunodeficiencies such as XLA remains poorly characterized. Traditionally, XLA patients have been considered resistant to EBV infection due to the absence of mature B cells, the virus’s primary latent reservoir [17,18,52]. This case report challenges that paradigm by documenting EBV persistence in peripheral leukocytes and tissues, despite a complete lack of circulating B cells. The clinical course, which includes EBV-positive T cell lymphoma and HLH, reveals a pathologic trajectory more commonly associated with CAEBV.

The comparative literature review in this study underscores key distinctions between XLA and CAEBV in terms of genetic architecture, immunopathogenesis, and clinical features [3,7,48]. Importantly, this case reopens the question of how EBV might enter and persist in the absence of B cells. Canonically, EBV latency is maintained in memory B cells, with reactivation occurring at mucosal sites, where epithelial cells are infected in a basolateral fashion [53,54]. The absence of this reservoir in XLA patients was previously thought to prevent persistent EBV infection [17,18,52]. Supporting this model, allogeneic bone marrow transplant studies have shown that transplanted patients acquire the donor’s EBV strain, reinforcing the role of B cells in viral persistence [55].

However, the detection of EBV in CD3+ T cells and in multiple tissues in our patient suggests alternative routes of viral entry and latency. One proposed mechanism is the transient infection of T or NK cells during the acute phase, as observed in infectious mononucleosis [56], where such infected cells typically undergo apoptosis, although rare conditions may permit their survival. Alternatively, trogocytosis has been suggested as a mechanism whereby T or NK cells acquire the EBV receptor CD21 from B cells at immunological synapses, thus enabling direct viral entry [57,58]. Although speculative, these mechanisms may help explain how EBV persists in non–B cell compartments, particularly in immunodeficient hosts.

Another notable feature is the persistent neutropenia, present at diagnosis and during subsequent hospitalizations. This complication, observed in 10–25% of XLA patients, has been attributed to BTK-related defects in myeloid cells or immune-mediated destruction [25,45,59,60,61]. It is noteworthy that neutropenia was resolved partially with immunoglobulin replacement, but recurred in the context of systemic inflammation and chemotherapy. Recent data on BTK inhibitors such as pirtobrutinib and zanub Tranebjaerg rutinib support the idea that BTK has broader roles in neutrophil survival and Fc receptor signaling, highlighting its relevance beyond B cell maturation [6,62,63].

What renders this case truly unique is the convergence of CAEBV-like features, HLH, EBV-positive T cell lymphoma, systemic EBV tissue infiltration, EBV peripheral load, and immune dysregulation within the well-defined genetic framework of XLA. To our knowledge, no other report has demonstrated EBV persistence by both qPCR and EBER-ISH in a patient with a confirmed *BTK* mutation and complete absence of mature B cells. This challenges current assumptions about EBV biology and extends the clinical spectrum of XLA. Clinicians should consider EBV in the differential diagnosis of XLA patients presenting with unexplained inflammation, cytopenias, or lymphoproliferative symptoms, even in the absence of classical serologic markers or plasma viral load. Further studies are warranted to elucidate noncanonical reservoirs and mechanisms of immune evasion in the context of humoral immunodeficiency.

## 5. Conclusions

This case challenges long-standing assumptions about the biology of EBV infection in the context of primary humoral immunodeficiencies. Despite the absence of mature B cells, the patient with genetically confirmed XLA developed EBV-positive T-cell lymphoma and HLH, demonstrating that EBV can persist and drive lymphoproliferative disease through noncanonical mechanisms. While EBV latency has classically been attributed to memory B cells, this report suggests that alternative reservoirs such as CD3+ T cells may sustain infection and pathogenesis in profoundly B cell-deficient hosts. These findings not only expand the clinical phenotype of XLA but also provoke reconsideration of EBV tropism, immune evasion, and viral-host dynamics in IEIs.

In summary, this case illustrates how clinical and laboratory features classically attributed to CAEBV can emerge in patients with underlying IEIs like XLA, challenging current classifications. Ultimately, this case illustrates the biological adaptability of EBV and highlights the importance of maintaining high clinical suspicion for EBV-related pathology in XLA patients with systemic inflammation or lymphoid abnormalities. It reinforces the need for further mechanistic studies to elucidate viral behavior in non-classical immune contexts and to guide future therapeutic strategies.

## Figures and Tables

**Table 1:** Patient laboratory results.

Test	Lab Findings at Diagnosis	Lab Results After Treatment
Lymphocyte subsets (cells/μL)		
CD3+ (1200–2600)	909.34 (49.4%)	1827
CD3+ CD8+ (370–1100)	371.76 (20.2%)	698
CD3+ CD4+ (650–1500)	529.48 (28.7%)	1096
CD16+ CD56+ (100–480)	745.64 (40.5%)	155
CD19+ (270–860)	7.39 (0.4%)	4
Blood cytometry		
Hemoglobin (Hb) (11.5–13.5 g/dL)	9.3 g/dL	13.6
Hematocrit (HCT) (35–40%)	29%	41.5
Platelets (150,000–450,000/µL)	136,000/µL	524,000
White blood cells (WBCs) (4500–13,500/µL)	1900/µL	
Neutrophils (40–70%)	7.6%	
Lymphocytes (20–40%)	46%	
Monocytes (2–10%)	47.7%	
Eosinophils (1–6%)	0.1%	
Reticulocytes (0.5–1.0%)	2.6%	
Immunoglobulins (mg/dL)		
IgG (608–1572)	105	920
IgA (45–236)	6.25	6.69
IgM (52–242)	17.30	4.54
IgE (0.98–57)	<18.20	<19.48
Viral panel (U/mL)		
EBV Viral capsid antigen IgG (VCAG) (Negative: <20; Gray Zone: 20–40; Positive: >40)	Negative	
VCAM	Negative	
EAD (Negative: <10; Gray Zone: 10–40; Positive: >40)	Negative	
EBNA	Negative	
EBV viral load		
Leukocytes (Negative)	640 copies/μg of DNA	
Plasma (Negative)	Negative	

**Table 2:** Chronological presentation of clinical manifestations.

Clinical Condition/Disease	Treatment
Vesicular dermatosis and cellulitis with persistent fever	Dicloxacillin
Bruton’s agammaglobulinemia (XLA) and pancitopenia	Monthly subcutaneous immunoglobulin 545 mg/kg.
Sudden neurological deterioration and right hemiparesis	Fronto-temporoparietal decompressive craniectomy
Chickenpox infection and atelectasis due to fungal infection	Voriconazole
Monoclonal lymphoproliferative process (T cell lymphoma)	Systemic chemotherapy: vincristine, daunorubicin, etoposide, Ara-C.
Neutropenic colitis and septic shock	Supportive care
Papulovesicular lesions and necrosis on the forearm	Acyclovir (1500 mg/day)
Pneumonia due to bocavirus (HBoV) and respiratory syncytial virus (RSV)	Supportive care
Episodes of febrile neutropenia	Supportive care
Hemophagocytic lymphohistiocytosis (HLH)	HLH-2004 protocol (Etoposide, dexamethasone, and cyclosporine A)
Epstein-Barr virus (EBV) infection	Not specified (diagnosis confirmed by PCR and EBER-ISH)
Confirmed *BTK* mutation	Not applicable
SARS-CoV-2 infection (COVID-19)	Supportive care

## Data Availability

All weighted de-identified data used here are included in this study. The datasets used and/or analyzed during the current study are available from the corresponding author on reasonable request. This literature review did not generate or analyze any new datasets. The study synthesized findings from existing published literature, all of which are cited within the manuscript.

## Supplementary Materials

The following supporting information can be downloaded at: https://www.mdpi.com/article/10.3390/jpm15080365/s1, Figure S1: Sanger sequencing and Western blotting. (A) Sanger sequencing of the index patient identifying the single nucleotide variant (CGA>TGA) in codon 520 of the *BTK* gene, consistent with the exome sequencing results. The patient marked with R520* corresponds to a sample of the uncle of our index patient and XLA1-6 to other individuals with XLA diagnosis. (B) Western blot shows no expression of BTK protein in a cohort of patients with X-linked agammaglobulinemia. GAPDH was used as a housekeeping gene. Table S1: Clinical spectrum and immunological characteristics of patients with XLA: case reports and cohort evidence (2014–2025). The table includes both classical and atypical presentations, including HLH and EBV-associated findings. BTK mutation types and therapeutic responses are listed when available. Table S2: Clinical spectrum and immunological characteristics of patients with CAEBV: case reports and cohort evidence (2014–2025). Table S3: Representative Laboratory Abnormalities Associated with CAEBV and XLA [64,65,66,67,68,69,70,71,72,73].
