# Supplementary material for: EBV-Driven HLH and T Cell Lymphoma in a Child with X-Linked Agammaglobulinemia: A Genetically Confirmed Case Report and Literature Review
Source: J Pers Med. 2025 Aug 9;15(8):365. doi: 10.3390/jpm15080365 (PMC12387490; doi:10.3390/jpm15080365)
Supplement: Supplementary file 1 [file jpm-15-00365-s001.zip › jpm-3708733-supplementary.pdf]

Supplementary Material

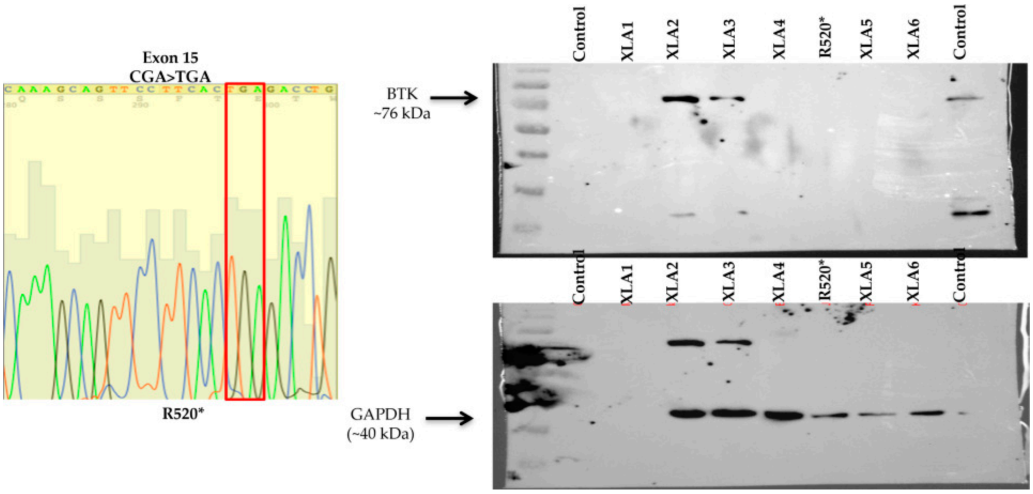

Supplementary Figure S1. The following supporting information can be downloaded at: https://www.mdpi.com/article/doi/s1, Figure S1: Sanger sequencing and western blotting. (A) Sanger sequencing of the index patient identifying the single nucleotide variant (CGA>TGA) at position 520 of the *BTK* gene, consistent with the exome sequencing results. The patient marked with R520* corresponds to a sample of the uncle of our index patient and XLA1-6 to other individuals with XLA diagnosis. (B) Western blot shows no expression of BTK protein in a cohort of patients with X-linked agammaglobulinemia. GAPDH was used as a housekeeping gene.

Supplementary Table S1. Clinical spectrum and immunological characteristics of patients with XLA: case reports and cohort evidence (2014–2025). The table includes both classical and atypical presentations, including HLH and EBV-associated findings. *BTK* mutation types and therapeutic responses are listed when available.

Clinical Manifestations	Cellular Deficiencies	Infections	Treatment	*BTK* Mutation	Ref.	Type of analysis
Proteinuria, microscopic hematuria, persistent nephritic findings in twin boys with XLA	Absent B cells (0%), low immunoglobulins	Not reported, focused on renal pathology	IVIG, antibiotics, interferon	c.119A>G (p.Tyr40Cys) in both cases	*Wan et al. 2024 [1]*	*Case report (n=2) twins*
Pulmonary alveolar proteinosis, recurrent respiratory infections, diarrhea	B cell deficiency (0.03%), very low IgG, IgM, IgA	No pathogens reported	IVIG	c.1751-1G>A	*Zhang et al. (2024) [2]*	*Case report (n=1)*
Recurrent abscesses, otitis media, rhinosinusitis, gastroenteritis, mouth ulcers, allergic rhinitis	Evolving B cell depletion, mild neutropenia, low phagocytic index, low IgM	*Staphylococcus aureus, Streptococcus pneumoniae, Salmonella sp., SARS-CoV2*	IVIG, antibiotics	c.1843C>T, p.Arg615Cys	*Markocsy et al. 2024 [3]*	*Case report (n=1)*
Recurrent pneumonia, otitis, skin infections,	Very low B cells (0–1%), low IgG, IgA, IgM	*Streptococcus pneumoniae,* SARS-CoV-2	IVIG, antibiotics	Multiple mutations across *BTK*, 5 novels	*Chear et al. 2023 [4]*	*Cohort study (n=22)*

arthritis, meningitis, COVID-19				(e.g., c.1181C>A, c.1559G>T)		
Recurrent diarrhea, abdominal pain, chronic mastoiditis, irritable bowel syndrome	Absent B cells (0%), undetectable IgG/IgA/IgM, thrombocytopenia	Fungal (not specified)	IVIG, steroids, vedolizumab, nutritional support	c.1558C>T (p.Arg520*)	*Deng et al. 2023 [5]*	*Case series (n=3)*
Severe pneumonia, pneumothorax	Low IgG, absent IgA/IgM	COVID-19 (SARS-CoV-2)	IVIG, dexamethasone, remdesivir, meropenem, Sotrovimab	*BTK* c.215_216insA (frameshift insertion)	*Rise et al. 2022 [6]*	*Case report n=1*
Meningitis at 5 months, skin lesions, ecthyma gangrenosum	Absence of B cells, neutropenia (ANC=0)	*Streptococcus pneumoniae*	IVIG, ceftriaxone, cefazolin, cephalexin,	Not specified	*Kadden et al. 2021 [7]*	*Case report n=1*
Chronic diarrhea, ileitis, malabsorption, IBD-like symptoms	Absence of B cells, low IgG/IgA/IgM	None reported (inflammatory etiology)	IVIG, steroids, infliximab, vedolizumab, ustekinumab	c.1138C>T (p.Q380*), c.1673_1680delAATTTCCA (p.Lys558fsX), c. 1697C> T (p.Pro566Leu)	*Khan et al. 2021 [8]*	*Case series n=3*
Sepsis, oral ecthyma gangrenosum, neutropenia, cleft lip sequelae	Neutropenia (1%), absence of B cells (0.4%-0.6%), low immunoglobulins	*Pseudomonas aeruginosa*	IVIG, ceftazidime, acyclovir, gentamicin, debridement	c.862C>T (R288W) in SH2 domain	*Lin et al. 2020 [9]*	*Case report (identical twins)*
Allergic rhinitis	CD19+ B cells (2-7%), high IgE, normal IgG/IgA, partial *BTK* expression, mildly deficient memory B cells	None reported (inflammatory profile)	Antihistamines, corticosteroids	c.82C>T (p.Arg28Cys) in PH domain	*Cinicola et al. 2020 [10]*	*Case report n=1*
**Pseudomonas sepsis, Shanghai fever, ecthyma gangrenosum, recurrent HLH, bronchiectasis, pericarditis, empyema, arthritis, colitis, hepatocellular carcinoma (rare), Mohr-Tranebjaerg syndrome**	Absent/low CD19+ B cells (0-1.3%), hypogammaglobulinemia, reduced *BTK* expression, recurrent HLH in one case	*Pseudomonas aeruginosa*, *Streptococcus pneumoniae*, Campylobacter, Salmonella, rotavirus	IVIG, antibiotics, supportive care, prenatal diagnosis for early intervention	Missense, splice-site, nonsense, large deletions, 6 novel mutations: c.1562A>T, c.1132T>C, c.895-2A>G, c.504G>T, c.910T>G, and c.1957delG	*Yeh et al., 2020 [11]*	*Cohort study (n=19)*

**(DDON/MTS) with ataxia and hearing loss**						
Purulent pericarditis, cardiac tamponade	Absent B cells (0.12%), low IgG/IgA/IgM, high neutrophils	*Streptococcus pneumoniae*	IVIG, piperacillin/tazobactam, pericardial drainage	Deletion of exons 8-9-10	*Martignani et al. 2020 [12]*	*Case report (n=1)*
Ecthyma gangrenosum, sepsis, recurrent pyogenic infections, recurrent HLH episodes	Absent B cells, pancytopenia during HLH	*Pseudomonas aeruginosa*	IVIG, corticosteroids, supportive care for HLH	c.1632-1G>A (splice site, novel)	*Han et al. 2019 [13]*	*Case report (n=1)*
Atopic dermatitis without IgE elevation, recurrent respiratory infections	Absent B cells (0.2%), low IgG, IgA, IgM, IgE	*Staphylococcus aureus*	Topical steroids, hydrocortisone butyrate	c.186delGAAA (frame-shift mutation)	*Yamazaki et al. 2019 [14]*	*Case report (n=1)*
Diarrhea (intermittent/chronic), abdominal pain, gastroesophageal reflux disease, gastroenteritis, colitis, IBD-like symptoms	Low to absent B cells, hypogammaglobulinemia	Rotavirus, adenovirus, *Giardia lamblia*, *C. difficile* (in some cases)	IVIG, corticosteroids, nutritional support	Mostly missense, some nonsense/frameshift c.653del (p.Lys218Argfs*11) c.1567-14T>A, c.1589T>C (p.Leu486Pro) c.43G>A (p.Arg28His) c.1565T>C (p.Leu522Pro) c.41C>A (p.Ser14Tyr)	*Barmettler et al. 2017 [15]*	*Cohort study (n=200, USIDNet database)*
Respiratory tract infections (bronchitis, pneumonia), chronic diarrhea, IBD	Low or absent B cells, low IgG, IgA, IgM	*Campylobacter jejuni*, *Giardia lamblia*, *E. coli*, *Salmonella sp.* rotavirus, *Ascaris lumbricoides*	IVIG, antibiotics, dietary management	Not specified	*Pac et al. 2017 [16]*	*Cohort study (n=44)*
Sepsis (culture), enteroviral meningoencephalitis, seizures, motor regression, ataxia	Absent B cells, neutropenia, low IgG, IgA, IgM	Enterovirus (suspect of coxsackievirus B5)	IVIG, interferon-α2b, cidofovir, pocapavir, brain biopsy diagnosis	c.763C>T (R255X)	*Bearden et al. 2016 [17]*	*Case report (n=1)*
Pneumonia, bronchitis, otitis, naso sinusitis, persisting diarrhea, CNS	Variable, most had absent B cells (<2%) and low IgG, IgA,	No pathogens reported	IVIG	84 mutations (include large deletions, missense, frameshift, splicing and nonsense	*Chen et al. 2016 [18]*	*Cohort study (n=174; retrospective analysis)*

infections, deep-seated infections, aseptic arthritis, skin infections	IgM, in a few cases neutropenia and thrombopenia		mutation) and 45 novel mutations	

Supplementary Table S2. Clinical spectrum and immunological characteristics of patients with CAEBV: case reports and cohort evidence (2014–2025).

Clinical manifestations	Cellular manifestations	Infections	Treatment	Mutations	Ref.	Type of analysis
Recurrent diarrhea, fever, abdominal pain; mimics IBD	EBER+ lymphocytes in colon mucosa; CD4+, CD8+ T-cell count low	EBV DNA ($4.93x10^2$ copies/mL blood, $6.34x10^4$ in pharyngeal secretions)	Dexamethasone, acyclovir	Not specified	*Liang & Qu, 2025 [19]*	Case report
Abdominal pain, bloody diarrhea, HLH; GI perforations in some cases, systemic manifestations (fever, lymphadenopathy, hepatosplenomegaly)	Lymphoproliferati on	EBV DNA (median $1.56x10^4$ copies/mL)	Chemotherapy (GLIDE, GED), PD-1 antibody, HSCT	Clonal and polyclonal TCR rearrange ments	*Shen et al., 2023 [20]*	Case series (7 cases)
**Hydroa vacciniforme-like lymphoproliferative disorder (HV-LPD), oral ulcers, CNS symptoms, coronary/cerebral aneurysms**	EBER+ T-cells (TCRγ+); CD3+, CD8+, CD30+, GRB+, TIA1+	Whole blood EBV DNA up to 2 ×106/mL; CSF 3.1×10^5 copies/mL	HSCT, dexamethasone, methotrexate, hydrocortisone, Bortezomib + valganciclovir,	TCR-γ and β rearrange ments in CSF	*Yi et al., 2023 [21]*	Case report
Acute liver failure, fever, abdominal pain, HLH (suspect)	Lymphocyte infiltration in liver CD8+, EBER+, CD20-, CD56-	EBV DNA 3.4×10^5 copies/μg DNA in explanted liver; 2.1×10^3 copies/μg in blood	HSCT, steroids, etoposide	TCR-γ in CD8+ T-cells	*Nakajima et al., 2022 [22]*	Case report
Subcutaneous nodules, fever, splenomegaly	EBER+ T-cells in skin and subcutaneous adipose tissue; CD3+, CD4+, CD8+, TIA-1+, GRB+	EBV DNA $1.42x10^6$ copies/mL	HSCT after chemotherapy	Not specified	*Luo et al., 2022 [23]*	Case report
Generalized myositis, erythema, subcutaneous	EBER+ NK/T lymphoma; CD3+, CD56+, CD4-/CD8-	EBV DNA $5.88x10^5$ (serum), $1.73x10^5$ in NK	Glucocorticoids, tacrolimus, ganciclovir, P-GEMOX	Not specified	*Shi et al., 2022 [24]*	Case report

nodules, lymphoma NK/T		DNA and 4.66×10^4 copies in B cells.	(gemcitabine, oxaliplatin and peaspargase), PD-1 antibody			
Fever, liver failure, hepatosplenomegaly, DIC, multiorgan failure	EBER+ CD3+ T-cells infiltrate	EBV DNA 1.5×10^5 copies/mL	Supportive care	Not studied	*Alotaibi et al., 2022 [25]*	Case report
**IM symptoms, HLH, fever, hepatosplenomegaly, lymphadenopathy, cytopenia, rash, seizures, DIC**	CD3+ > CD20+ cells, lymphoproliferation EBER +, inverted CD4/CD8 ratio	Median EBV-DNA 2.43×10^5 IU/mL in blood	Antivirals, corticosteroids, immunoglobulin, cyclophosphamide, cyclosporine A, etoposide, vincristine, and tacrolimus for HLH	Monoclonal TCR-γ and β rearrangements	*Lin et al., 2021 [26]*	Retrospective cohort (19 adults)
Fever, hepatosplenomegaly, HLH, rash, coronary dilatation	EBER+ cells in tissue; mostly T-cell type (16/25), NK-cell (9/25)	Median EBV-DNA of 9.9×10^6 copies/ml in PBMCs and 3.1×10^4 copies/ml in plasma	HSCT, HLH-94 Protocol	Not specified	*Luo et al., 2021 [23]*	Case series (25 pediatric cases)
Bilateral or unilateral coronary artery dilatation (CAD), hepatomegaly, splenomegaly, fever, skin rashes, HLH	Cytopenias, cells EBER+ in bone marrow, lymph nodes, and liver	Median EBV-DNA of 1.18×10^7 copies/ml in whole blood and 1.81×10^4 copies/ml in plasma	HSCT	Not specified	*Wei et al, 2021 [27]*	Case series (10 cases)
Cytopenias, HLH, NK or T-cell lymphoma, hydroa vacciniforme–like lymphoproliferative disease	T, T/NK, T/B-cell EBV+, cytopenia	Median EBV DNA 6.3×10^5 copies/mL in whole blood	HSCT, dexamethasone and etoposide, rituximab, bortezomib, ganciclovir, steroids	Not specified	*Saldaña et al., 2022 [28]*	Case series (57 cases)
Decreased vision, cough, uveitis, mitral and aortic valve insufficiency	EBV+ Lymphoid bone marrow infiltration (CD3+, CD5+, CD7+, CD2+, CD20-, CD56-)	EBV DNA 3.29×10^4 copies/mL in whole blood	HSCT, prednisone, ciclosporin A, ganciclovir	Not found in WES	*Xiao et al., 2020 [29]*	Case report
Severe pulmonary arterial hypertension, cardiac insufficiency, systemic vasculitis, fever hepatomegaly	Lymphoproliferation EBV+ EBER+ CD8+ T cells in lung; mild lymphoid infiltration	EBV DNA 4.6×10^6 copies/mL of plasma	Steroids and sildenafil, valaciclovir	Not reported	*Ba et al., 2019 [30]*	Case report

Fever, splenomegaly, lymphadenopathy, hepatitis, pulmonary arterial hypertension, cardiovascular symptoms	Low B, NK, CD4, and CD8 cells	EBV DNA 2.5×10^{4} copies/mL in serum	Not reported	Not specified	*Luo et al., 2018 [31]*	Case series (28 cases)
Hydroa vacciniforme, IM-like symptoms, liver dysfunction, hepatosplenomegaly	Colon infiltration of monoclonal T-cells EBV+	EBV DNA 5.7 x10$_{5}$ copies//µg of DNA	Etoposide, prednisolone, adriamycin, asparaginase	Not specified	*Kaneko et al., 2018 [32]*	Case report
Recurrent fever, weight loss, hematochezia, hepatosplenomegaly, skin nodules and lesions, fatal GI perforations	Systemic lymphoma T/NK EBV positive	EBV-positive T/NK-cell lymphoma confirmed in intestine and skin, *Salmonella typhi*	Supportive care, antibiotics, mesalazine and prednisone	Not specified	*Xiao et al., Medicine, 2016 [33]*	Case report
Hydroa vacciniforme-like, IM-like symptoms, pulmonary nodules, hepatosplenomegaly, erythema, GI symptoms, lymphoma	Cutaneous T-cell lymphoproliferative disorder EBV+, oral and blood clonal lymphoproliferation	EBV DNA 8.9×10^{4} copies/10^{6} cells; persistent EBV positivity	EPOCH, Capizzi therapy, cord blood transplantation	Clonal EBV-infected cells via Southern blot	*Saburi et al., 2016 [34]*	Case report
Persistent fever, cardiovascular complications	Not specified	EBV DNA 2.8×10^{4} copies/mL; recurrent viremia	Medrol, Leflunomide	Not specified	*Jiang et al., 2015 [35]*	Case report

Supplementary Table S3. Representative Laboratory Abnormalities Associated with CAEBV and XLA.

	**Clinical laboratory findings**	**Ref.**
CAEBV	Elevated transaminases (ALT: 1,806 U/L; AST: 1,444 U/L), CK-MB: 28 U/L; elevated ferritin: 91,893 µg/L	Wei et al., 2022 [27]
CAEBV	Elevated transaminases (AST: 708 U/L; ALT: 155 U/L); ALP: 1,111 U/L; BUN: 17.4 mmol/L; creatinine: 135 µmol/L	Alotaibi et al., 2022 [25]
XLA	Elevated ferritin: 1,214 ng/mL; triglycerides: 344 mg/dL; soluble IL-2 receptor alpha (sIL-2Ra) >5,000 pg/mL	Han et al., 2019 [13]
XLA	Slightly elevated LDH: 238 U/L; C-reactive protein (CRP): 8.1 mg/L	Wan et al., 2024 [1]

References

[1] Wan S, Cao M, Zou J, et al. Case report of renal manifestations in X-linked agammaglobulinemia. *Front Immunol*; 15. Epub ahead of print 2024. DOI: 10.3389/fimmu.2024.1376258.

[2] Zhang T, Li M, Tan L, et al. Pulmonary alveolar proteinosis induced by X-linked agammaglobulinemia: A case report. *World J Clin Cases* 2024; 12: 1644–1648.

[3] Markocsy A, Kapustová D, Čereš A, et al. Atypical Manifestation of X-linked Agammaglobulinemia - the Importance of Genetic Testing. *Acta Medica (Hradec Kralove)* 2024; 67: 60–63.

[4] Chear CT, Ismail IH, Chan KC, et al. Clinical features and mutational analysis of X-linked agammaglobulinemia patients in Malaysia. *Front Immunol*; 14. Epub ahead of print 2023. DOI: 10.3389/fimmu.2023.1252765.

[5] Deng F, Wang H, Wang X. Chronic Diarrhea with Villous Blunting of the Small Intestine Under Capsule Endoscopy in Common Variable Immunodeficiency and X-Linked Agammaglobulinemia: A Case Series. *J Asthma Allergy* 2023; 16: 997–1006.

[6] Rise N, Touborg T, Lundsted DH, et al. Case report: Evolution of pulmonary manifestations and virological markers in critical COVID-19 infection in Bruton's agammaglobulinemia. *Front Immunol* 2022; 13: 1–7.

[7] Kadden D, Fowler G, Engel E, et al. Streptococcal pneumonia meningitis as an initial presentation of X-linked agammaglobulinemia: A case report and discussion. *JACEP Open* 2021; 2: 10–13.

[8] Khan F, Person H, Dekio F, et al. Crohn's-like Enteritis in X-Linked Agammaglobulinemia: A Case Series and Systematic Review. *Journal of Allergy and Clinical Immunology: In Practice* 2021; 9: 3466–3478.

[9] Lin SC, Chiang BL, Lee YJ, et al. Pseudomonas aeruginosa sepsis presenting as oral ecthyma gangrenosum in identical twins with Bruton tyrosine kinase gene mutation: Two case reports and review of the literature. *Journal of Microbiology, Immunology and Infection* 2020; 53: 1030–1034.

[10] Cinicola B, Uva A, Leonardi L, et al. Case Report: A Case of X-Linked Agammaglobulinemia With High Serum IgE Levels and Allergic Rhinitis. *Front Immunol* 2020; 11: 1–6.

[11] Yeh YH, Hsieh MY, Lee WI, et al. Distinct Clinical Features and Novel Mutations in Taiwanese Patients With X-Linked Agammaglobulinemia. *Front Immunol*; 11. Epub ahead of print 2020. DOI: 10.3389/fimmu.2020.02001.

[12] Martignani C, Massaro G, Bruno AG, et al. Acute primary purulent pericarditis in an adult patient with unknown X-linked agammaglobulinemia. *Immunobiology*; 225. Epub ahead of print 2020. DOI: 10.1016/j.imbio.2019.10.010.

[13] Han SP, Lin YF, Weng HY, et al. A novel BTK gene mutation in a child with atypical X-linked agammaglobulinemia and recurrent hemophagocytosis: A case report. *Front Immunol* 2019; 10: 1–7.

[14] Yamazaki E, Kikuchi K, Sasahara Y, et al. Atopic dermatitis without serum immunoglobulin E elevation or loss-of-function filaggrin gene mutation in a patient with X-linked agammaglobulinemia. *Journal of Dermatology* 2020; 47: 58–60.

[15] Barmettler S, Otani IM, Minhas J, et al. Gastrointestinal Manifestations in X-linked Agammaglobulinemia. *J Clin Immunol* 2017; 37: 287–294.

[16] Pac M, Bernatowska EA, Kierkuś J, et al. Gastrointestinal disorders next to respiratory infections as leading symptoms of X-linked agammaglobulinemia in children - 34-year experience of a single center. *Archives of Medical Science* 2017; 13: 412–417.

[17] Bearden D, Collett M, Quan PL, et al. Enteroviruses in X-Linked Agammaglobulinemia: Update on Epidemiology and Therapy. *Journal of Allergy and Clinical Immunology: In Practice* 2016; 4: 1059–1065.

[18] Chen XF, Wang WF, Zhang YD, et al. Clinical characteristics and genetic profiles of 174 patients with X-linked agammaglobulinemia: Report from Shanghai, China (2000-2015). *Medicine (United States)*; 95. Epub ahead of print 2016. DOI: 10.1097/MD.0000000000004544.

[19] Liang M, Qu J. Chronic active Epstein–Barr virus colitis, a rare cause of recurrent diarrhea in an immunocompetent female: a case report. *BMC Infect Dis* 2025; 25: 50.

[20] Shen K, Shuai X, Li J, et al. Chronic active Epstein-Barr virus infection involving gastrointestinal tract with hemophagocytic lymphohistiocytosis. *Ann Hematol* 2023; 102: 45–53.

[21] Yi T, Steinberg J, Olson S, et al. Chronic active Epstein–Barr virus and hydroa vacciniforme-like lymphoproliferative disorder in a pediatric patient complicated by fatal ruptured cerebral artery aneurysm. *Clin Case Rep*; 11. Epub ahead of print June 2023. DOI: 10.1002/ccr3.7436.

[22] Nakajima K, Hiejima E, Nihira H, et al. Case Report: A Case of Epstein-Barr Virus-Associated Acute Liver Failure Requiring Hematopoietic Cell Transplantation After Emergent Liver Transplantation. *Front Immunol*; 13. Epub ahead of print 28 January 2022. DOI: 10.3389/fimmu.2022.825806.

[23] Luo YH, Yang J, Wei A, et al. Haploidentical hematopoietic stem cell transplantation for pediatric patients with chronic active Epstein–Barr virus infection: a retrospective analysis of a single center. *World Journal of Pediatrics* 2021; 17: 626–636.

[24] Shi S, Li L, Pan C, et al. Case report: Systemic muscle involvement as the primary clinical manifestation of chronic active Epstein–Barr virus infection: A case-based review. *Front Immunol*; 13. Epub ahead of print 7 October 2022. DOI: 10.3389/fimmu.2022.1027859.

[25] Alotaibi Y, Albogami M, Alsaedy A, et al. A Lethal Manifestation of Chronic Active Epstein-Barr Virus Infection: A Case Report. *Cureus*. Epub ahead of print 11 October 2022. DOI: 10.7759/cureus.30158.

[26] Lin J, Wu H, Gu L, et al. Clinicopathologic findings of chronic active Epstein–Barr virus infection in adults: A single-center retrospective study in China. *Clin Exp Med* 2021; 21: 369–377.

[27] Wei A, Ma H, Zhang L, et al. Clinical analysis of chronic active EBV infection with coronary artery dilatation and a matched case–control study. *Orphanet J Rare Dis*; 16. Epub ahead of print 1 December 2021. DOI: 10.1186/s13023-021-01689-5.

[28] Davila Saldana BJ, John T, Bonifant C, et al. High risk of relapsed disease in patients with NK/T-cell chronic active Epstein-Barr virus disease outside of Asia. *Blood Adv* 2022; 6: 452–459.

[29] Xiao H, Hu B, Luo R, et al. Chronic active Epstein–Barr virus infection manifesting as coronary artery aneurysm and uveitis. *Virol J*; 17. Epub ahead of print 1 December 2020. DOI: 10.1186/s12985-020-01409-8.

[30] Ba H, Xu L, Peng H, et al. Chronic active Epstein-Barr virus infection with systemic vasculitis and pulmonary arterial hypertension in a child. *Front Pediatr*; 7. Epub ahead of print 2019. DOI: 10.3389/fped.2019.00219.

[31] Luo L, Wang H, Fan H, et al. The clinical characteristics and the features of immunophenotype of peripheral lymphocytes of adult onset chronic active Epstein-Barr virus disease at a Tertiary Care Hospital in Beijing. *Medicine (United States)*; 97. Epub ahead of print 1 March 2018. DOI: 10.1097/MD.0000000000009854.

[32] Kaneko H, Taniwaki M, Matsumoto Y, et al. An adult-onset case of chronic active Epstein-Barr virus infection with fulminant clinical course. *Journal of Infection and Chemotherapy* 2018; 24: 479–482.

[33] Xiao HJ, Li J, Song HM, et al. Epstein-Barr virus-positive T/NK-cell lymphoproliferative disorders manifested as gastrointestinal perforations and skin lesions a case report. *Medicine (United States)*; 95. Epub ahead of print 2016. DOI: 10.1097/MD.0000000000002676.

[34] Saburi M, Ogata M, Satou T, et al. Successful cord blood stem cell transplantation for an adult case of chronic active epstein-barr virus infection. *Internal Medicine* 2016; 55: 3499–3504.

[35] Jiang L, Gu ZH, Yan ZX, et al. Exome sequencing identifies somatic mutations of DDX3X in natural killer/T-cell lymphoma. *Nature Genetics 2015 47:9* 2015; 47: 1061–1066.
